# 

**Published:** 1887-04-09

**Authors:** 


### The Hospital.

**An Institution, Family, and Parochial Journal of Hospitals, Asylums, and all Agencies for the Care of the Sick, Criticism and News.**

SATURDAY, APRIL 9TH, 1887.

### THE LITERARY PRIZE.

A PRIZE of **Two Guineas** will be given every month for the best story or incident based upon hospital, or asylum, or student life, or any incident connected with the professions of medicine or nursing.

### HOSPITAL HOUSEKEEPING.

FORMS and full particulars of the Quilter Prizes under this head (see page 355 for 19th Feb.) may be had on application to the Editor of THE HOSPITAL, Norfolk House, W.C. All competitive essays to be sent in by 30th April, 1887. Prizes, £10 10*s*. and £5 5*s*.

THE CHILDREN'S CORNER and AMUSEMENTS WITH FIT will be found on page vi of this week's issue.

### THE IN- AND OUT-PATIENTS' HOSPITAL DIARY.

This Diary is so arranged as to make some portion of it appear every week, and the whole in each monthly part. It has been very difficult to compile, and corrections will at all times be gratefully received. It has been suggested that similar information about the larger Provincial and Scotch Hospitals would be useful to many readers. We should be glad to hear if this is felt to be the case.

### GENERAL HOSPITALS.—*continued.*

| Hospitals and Terms of Admission. | Physicians, Surgeons, and Medical Officers, with Days and Hours of Attendance. |
|---|---|
| St. Mary's Hospital, Cambridge Place, Paddington, W. Sec., Mr. Pietro Michelli<br>In-patients are admitted by letter of recommendation. Urgent cases and accidents free at all times. Out-patients require a letter. Electrical treatment Tu. and F. 2 | *In-Physicians*, Sir Edward Sieveking, Tu. F. 1.45; Drs. Broadbent, M. Th. 1.45; Cheadle, W. 1.45, S. 9.30<br>*In-Surgeons*, Messrs. Norton, M. Th. 1.45; Owen, Tu. F. 1.45; H. Page, W. S. 1.45<br>*Out-Physicians*, Drs. D. B. Lees, W. S.; S. Phillips, M. Th; R. Macguire, Tu. F. Hour, 1.30<br>*Out-Surgeons*, Messrs. W. Pye, M. Th.; A. J. Pepper, Tu. F.; A. Q. Silcock, W. S. Hour, 1.30 |
| St. Thomas's Hospital, Westminster Bridge Road, S.E. Steward, Mr. F. Walker<br>Accidents and emergencies free at all times. Other cases by Governor's letter, but, where unobtainable, application should be made to the Steward by letter, or personally between 10 and 4. Vaccination on W. at 11.30 | *In-Physicians*, Drs. Bristowe, Tu. F.; Stone, M. Th.; Ord, M. Th.; J. Harley, Tu. F.; Gervis, Tu. F. Hour, 2<br>*In-Surgeons*, Messrs. Sydney Jones, Tu. F.; Croft, M. Th.; Sir W. MacCormac, M. Th. Hour, 2<br>*Out-Physicians*, Drs. Payne, Tu. F.; Sharkey, M. Th.; Gulliver, W. S. Hour, 1.30<br>*Out-Surgeons*, Messrs. Clutton, Tu. F.; Anderson, W. S.; Pitts, M. Tu. Hour, 1.30 |
| Tottenham Training Hospital, Tottenham, N. Director, Dr. Laseron<br>Free. Accidents and emergencies admitted at all times | *In-Physician*, Dr. Rasch, Tu. F. 3 to 5<br>*In-Surgeons*, Drs. Lichtenberg, M. Th. 3 to 5; E. H. May, M. Th. 3 to 5<br>*Out-Surgeon*, Dr. Lloyd Smith, M. W. 11 to 2; *Men* only, W. 7 to 8 p.m. |
| University College Hospital (or North London), Gower Street, W.C. Sec., Mr. N. H. Nixon<br>Accidents and emergencies free. All other cases by Governor's letter of recommendation | *In-Physicians*, Drs. W. Fox, Tu. Th. 2, S. 9.30; Ringer, M. W. F. 1.30; Bastian, M. 2.30, W. 10, F. 2.30; F. Roberts, Tu. 1.15, Th. 1.30, S. 9.30<br>*In-Surgeons*, Messrs. B. Hill, Tu. 2.15, W. 2, F. 2.15; C. Heath, M. W. F. 2; M. Beck, Tu. Th. S. 2<br>*Out-Physicians*, Drs. Gowers, W. S.; Poore, Tu. F.; Barlow, M. Th. Hour, 1.30<br>*Out-Surgeons*, Messrs. Barker, M. Th.; Godlee, Tu. F.; Horsley, W. S. Hour, 1.30 |
| West London Hospital, Hammersmith Road, W. Sec. and Supt., Mr. R. J. Gilbert<br>Accidents and emergencies free at all times. Other cases require a letter of recommendation. New cases must attend at 1.30 | *In-Physicians*, Drs. G. Rogers, D. W. Hood, F. Drewitt<br>*In-Surgeons*, Messrs. C. Keetley, F. Edwards, W. B. Clarke<br>*Out-Physicians*, Drs. Herringham, M. Th.; Ball, Tu. F.; S. Taylor, W. S. Hour, 2<br>*Out-Surgeons*, Messrs. Ballance, Tu. F.; Weiss, M. Th.; Wainewright, W. S. Hour, 2 |
| Westminster Hospital, Broad Sanctuary, S.W. Sec., Mr. S. M. Quennell<br>Accidents and urgent cases free at all times. Other cases require a letter of recommendation | *In-Physicians*, Drs. Sturges, M. Th. S.; Allchin, M. Tu. F.; Donkin, Tu. W. S. Hour, 1.30<br>*In-Surgeons*, Messrs. Cowell, M. Tu. Th.; Davy, M. Tu. F.; Macnamara, M. W. S. Hour, 1.30<br>*Out-Physicians*, Drs. De H. Hall, M. Th.; A. H. Bennett, Tu. F.; Murrell, W. S. Hour, 1.30<br>*Out-Surgeons*, Messrs. Cooke, M. Th.; Bond, Tu. F.; Barrow, W. S. Hour, 1.30 |

### II.—SPECIAL HOSPITALS.

### CANCER.

| | |
|---|---|
| Cancer Hospital, Brompton, S.W. Sec., Mr. W. H. Hughes<br>Entirely free to both in- and out-patients | *In- and Out-Surgeons*, Drs. A. Marsden, W.; H. Snow, M.; F. A. Purcell, F.; Messrs. F. B. Jessett, W.; C. Stonham, Th.; C. Jennings, Tu.; W. H. Elam, S. Hour, 2 |
| London Hospital, Whitechapel Road, E. | Daily, 1.30. *See* General Hospitals |
| Middlesex Hospital, Berners Street, W. Admission free | *In-Surgeons*, Messrs. Hulke, Lawson, and Morris<br>*Out-Surgeon*, Mr. Morris, Th. 1.30 |
| St. Saviour's Hospital, Osnaburg Street, N.W. Sec., Mr. E. Poitiers<br>By Governors' letters | *In- and Out-Physician*, Dr. A. Kennedy, 10.30 and 4 daily |

### CHILDREN'S DISEASES.

| Hospitals and Terms of Admission. | Physicians, Surgeons, and Medical Officers, with Days and Hours of Attendance. |
|---|---|
| Alexandra Hospital for Children, 18, Queen Sq., W.C. Sec., Mrs. Marsh<br>For hip disease | *Physician*, Dr. Steavenson<br>*Surgeons*, Messrs. H. Marsh Tu. F.; A. Bowlby, M. Th. Hour, 4.30<br>(No out-patients) |
| All Saints' Children's Hospital, 4, Margaret Street, W.<br>For chronic joint diseases | *Surgeon*, Mr. N. Smith<br>(No out-patients) |
| Belgrave Hosp. for Children, 77 & 79, Gloucester Street, Pimlico. Hon. Secs., Capt. Stopford, and Rev. J. Storrs<br>By letters; accidents free | *In- and Out-Physicians*, Drs. W. Hope and W. Ewart, M. Th. 9<br>*In- and Out-Surgeons*, Messrs. C. T. Dent and Margerison, Tu. F. 9 |
| Charing Cross Hospital, West Strand, W.C. | *Out-Physician*, Dr. Nursby, W. S. 1.30 |
| Cheyne Hospital for Sick and Incurable Children, 46, Cheyne Walk, Chelsea, S.W. Sec., Miss D. Evans | *Physician*, Dr. G. V. Poore<br>*Surgeons*, Messrs. T. P. Bartlett and J. Macready attend alternate days in afternoons. (No out-patients) |
| East London Hospital for Children and Dispensary for Women, Glamis Road, Shadwell, E. Sec., Mr. Ashton Warner<br>By Governor's letter. Urgent cases and accidents are admitted free | *In-Physicians*, Drs. Donkin, M. Th.; Eustace Smith, T. F.; Crocker, F.<br>*In-Surgeons*, Messrs. Calder, W. F.; Parker, W. F.<br>*Out-Physicians*, Drs. Crocker, M.; Williams, Tu. Th.; Coutts, W. F. Hours, 1 and 3<br>*Out-Surgeon*, Mr. Dunn, Tu. F. 1, 3 |
| Evelina Hospital for Sick Children, Southwark Bridge Road, S.E. Sec., Mr. T. S. Chapman.<br>Free, but Governor's letter takes precedence. In-patients (boys) between 2 and 10, (girls) between 2 and 12 | *In-Physicians*, Drs. F. Taylor and J. Goodhart<br>*In-Surgeons*, Messrs. H. G. Howse and R. C. Lucas<br>*Out-Physicians*, Drs. N. Tirard, M. Th. 9; F. Willcocks, Tu. F. 9<br>*Out-Surgeons*, Messrs. C. J. Symonds, S. 9; G. H. Makins, W. 9 |
| German Hospital, Dalston Lane, Dalston, E. | *In-Physician*, Dr. Rasch, M. Th. 9 |
| Great Northern Central Hospital, Caledonian Rd. N. | *Physicians*, Drs. Murray and Barnes, W. 2.30 |
| Grosvenor Hospital for Women and Children, Vincent Sq., S.W. Hon. Secs., Mr. A. J. Ram and Miss Day<br>By letter or payment | *In- and Out-Physicians*, Drs. R. Gibbons and Steavenson, Tu. W. Th. S. 2<br>*In- and Out-Surgeons*, Messrs. Smythe and Parker, Tu. W. Th. S. 2 |
| Hospital for Sick Children, 48 and 49, Great Ormond St., W.C. Sec., Mr. S. Whitford<br>By Governors' letters. If without letter, the case of the applicant is investigated<br>In-patients between 2 and 12; Out-patients under 12 | *In-Physicians*, Drs. W. Cheadle, Tu. F.; Sturges, W. S.; Barlow, M. Th. Hours, 9 to 11<br>*In-Surgeons*, Messrs. H. Marsh, W. S.; E. Owen, W. S. Hours, 9 to 11<br>*Out-Physicians*, Drs. R. J. Lee and Abercrombie, M. Th.; Lubbock and Money, Tu. F. Hours, 8.30 to 10<br>*Out-Surgeons*, Messrs. Morgan and Pitts, W. S. 8.30 to 10 |
| King's College Hospital, Portugal Street, W.C. | *In- and Out-Physician*, Dr. T. C. Hayes, W. F. 1 |
| Middlesex Hospital, Berners Street, W.<br>Free. Children under 3 | *Out-Physician*, Dr. Duncan, W. S. 1.30 |
| North Eastern Hospital for Children, Hackney Road, E. Sec., Mr. A. Nixon<br>By free ticket or small payment | *In- and Out-Physicians*, Drs. W. Cayley, F. Turner, C. Semple, and W. Pasteur, M. W. Th. S. 1.30<br>*In- and Out-Surgeons*, Messrs. Waren Tay and Pollard, M. 1.30, F. 9 |
| Paddington Green Children's Hosp., Paddington Green. Sec., Mr. W. H. Pearce<br>Admission free; boys under 12; girls under 14 | *In- and Out-Physicians*, Drs. T. Lauder Brunton, Ogilvie, Tu. F.; G. Laycock, M. Th.; S. Phillips, W. S. Hour, 9.15<br>*In- and Out-Surgeons*, Messrs. W. W. Cheyne, M., S. Boyd, F. Hour, 9.15 |
| Royal Hospital for Children and Women, Waterloo Bridge Road, S.E. Sec., Mr. R. G. Kestin<br>Out-Patients pay 1*d.* on each attendance. Boys not admissible, as out-patients, over 12, or, as in-patients, over 8 | *In-Physicians*, Drs. Park, M. Th.; Gulliver, Tu. F.; Price, W. S. Hour, 2<br>*In-Surgeon*, Mr. W. H. A. Jacobson, M. Th. Hour, 2<br>*Out-Physicians*, Drs. Park, M. Th.; Dakin, W. F.; Hadden, Tu. S. Hour, 2<br>*Out-Surgeon*, Mr. E. O. Day, W. 2 |
| St. Mary's Hospital, Cambridge Place, Paddington | *Out-Physician*, Dr. M. Handfield Jones, M. Th. 1.30 |
| St. Thomas's Hospital, Westminster Bridge Road, S.E | *Out-Physician*, Dr. Cory, W. S. 1.30 |

### THE CHILDREN'S CORNER.

### Third Quarterly Prize Competition.

Began 2nd Apr., 1887 ; ends 25th June, 1887.

PRIZES.

FOR MOST CORRECT ANSWERS TO PUZZLES.

| | | | |
|---|---|---|---|
| 1st .. | .. Thirty Shillings | 3rd .. | .. Ten Shillings |
| 2nd .. | .. Twenty „ | 4th .. | .. Five „ |

FOR NEATEST ANSWERS TO PUZZLES.

Five Shillings.

FOR BEST LITTLE LETTERS.

Ten Shillings.

### Puzzles—Second Week.

No. 6. CONUNDRUM. Who can tell me why an oyster is a great paradox ?

No. 7. BURIED AUTHORS. A tent for soldiers and a carillon ; an engine to grind corn and a heavy weight ; that which moves with celerity ; to total, myself in a very short word, and a male descendant.

No. 8. SUBTRACTION. How can you take forty-five from forty-five, and yet leave forty-five ?

No. 9. ENIGMA.

My *first* is joyous, happy, pleased ;
My *second's* often hurled;
My *whole's* a man whose name is known
Throughout the wide, wide world.

### Letters—Second Week.

The Letter-subject for next week will be "Home."

### Results of Thirteenth Competition—Puzzles.

No. 67. GEOGRAPHICAL PUZZLE. Rugby ; remove the "g,' and we have "ruby."

No. 68. HIDDEN WORD. The answer here is "Doctor."

No. 69. CONUNDRUM. Night draws nigh when t (tea) is removed, for then *night* is *nigh.*

No. 70. BURIED POETS. Pope, Gray, Longfellow, Dryden.

No. 71. CHARADE. Wonder.

No. 72. ARITHMETICAL QUESTION. The total, a hundred, can be made by two or three contrivances, thus :—

| | | | | |
|---|---|---|---|---|
| 45 | 24 | 74 | $48.\dot{9}$ | |
| 36 | 65 | 5 | 37 | |
| 17 | — | 8 | 6 | $\dot{9}=\frac{9}{9}=1$ |
| — | 89 | 9 | 5 | |
| 98 | 3 | $\frac{2}{1}$ | 2 | |
| 2 | 1 | $\frac{6}{3}$ | 1 | |
| — | 7 | — | — | |
| 100 | — | 100 | 100 | |
| | 100 | | | |

*All right*—Isobel, Scrooge, P.S., Florrie ; *Five right*—Peeler, Tim, A. C. K., Mikado, Skye, Agatha, Ellie, P. M. ; *Four right*—Joe, Dot, Alsie, Little Mary.

### Letters.

The "holiday" letters have, I think, even eclipsed all former ones. Peeler writes an admirable account of his visit last summer to Germany and Switzerland ; Isobel spent hers at Scarborough ; Florrie describes that newly-discovered watering-place, Clacton-on-Sea, while Scrooge tells me of her visit to Llandudno and the Great and Little Ormes. Skye writes very amusingly of her holidays in the Isle of Man ; Mikado visited Kenilworth and Warwick, while Tim and Ellie enjoyed excursions nearer town. I am very pleased to welcome two new little correspondents, whose letters I subjoin.

DEAR MR. EDITOR,—I live in Somersetshire. Last summer holidays, I went to nearly all the "meets" of the stag hounds, and sometimes followed them over the hills, and in the combes. My birthday was in August, when I had a picnic on the shore at Hurtstone Point. We went through a large cave which is called The Hole. I used to go up on the hills to pick "worts," which make one's teeth very black : they are little blue berries. Once I went to the top of Dunkerry Beacon, which is the highest point of Exmoor.

I remain, yours truly, AGATHA, aged 10½.

Porlock, Somerset.

DEAR MR. EDITOR,—I am going to tell you about last summer holidays, which I spent at home. Bathing was very nice when the water was not too cold, but I hated having to duck my head under water. I tried to swim, but could not : at last with an oar I got on better. I bathed near a boat-house with my three sisters. Sometimes our French governess bathed too ; she looked very funny in the water. In the middle of our river are two very old boats. They are called "Lighters." They look like twin brothers standing beside each other. When we wanted to bathe over the other side, we could go and undress on the "Lighters," and then row over. I hope this year I shall get on better with my swimming. Dear Mr. Editor, if you like this letter, I shall be very pleased to write again, because I know my sister (who is a hospital nurse) would love to see my name in the paper.

Believe me, yours truly, MURIEL, aged 8¾.

Broom Hills, Rochford, Essex.

Marks : Five each, Skye, Florrie ; one each, Halford, Tim, Ellie, Mikado ,Scrooge, Isobel ,Peeler, Agatha.

### Results of Second Quarterly Competition.

PUZZLES.

First Prize, 30*s.*, won by A. C. K. Score 68. (Master Arthur Clifton Kelly, aged 10, Ellesmere, Gratwicke Road, Worthing.)

Second Prize, 20*s.*, won by Tim. Score 67. (Master Cecil Anstey, aged 10, 25, Dornton Road, Balham, S.W.)

Third Prize, 10*s.*, won by Mikado. Score 65. (Miss F. H. Gibson, aged 15, 12, Upper Tichborne Street, Leicester.)

Fourth Prize, 5*s.*, a tie between Scrooge (Miss Constance Mills, 7, Charles Street, Pendleton, Manchester, aged 13) ; and P. S. (Master Percival George Silley, 40, Warwick Gardens, Kensington, W.) Score 64 each. This prize has accordingly been divided.

LETTERS.

Letter-writing is an *ars in arte.* We have first to consider the grammatical composition, the synthesis, etc., and secondly the matter. Bearing in mind the general excellence of most of the letters, I have had some difficulty in adjudging this prize. In the treatment of a subject, Florrie and Skye both display much power, and the quiet humour of the latter is always a pleasing feature of her letters. I have therefore divided the prize, 10*s.*, as under :—

Skye, 16 marks (Miss Ella R. Jefferiss, 4, Milton Terrace, New Road, Chatham, aged 14).

Florrie, 16 marks (Miss Florence Rhoda Powell, 20, St. John's Villas, East Dulwich Green, aged 14).

NEATNESS.

The prize, 5*s.*, for general neatness in the papers sent in, has been won by

Ellie (Miss Jessie Harris, 44, Burgoyne Road, Brixton, aged 11).

I wish all could have won prizes, but as this was not possible, I trust that defeat will only stimulate those who have not, to renewed exertions in the new competition. The contest in the last was exceedingly close.

Answers must be addressed to the "PUZZLE EDITOR," THE HOSPITAL, Norfolk House, Norfolk Street, Strand, W.C., not later than Thursday next. The "Children's Corner" Coupon (see advertisement pages) must be enclosed.

### AMUSEMENTS WITH PROFIT.

### PRIZE COMPETITIONS.

### No. 23. Double Acrostic.

A city, 'twas built by Charlemagne the Great ;
In Prussia the scene of a furious fight ;
'Tis a state whose men named a London street ;
For battle renown'd—Pictavium's site.

What my primals spell, I ne'er before have ask'd,
My name you'll see, when finals are unmask'd.

### No. 24. Mathematical Question.

A charitable person proposed to distribute £6 among some hospitals. He ultimately divided the amount among two more institutions than he originally intended, and, by so doing, the share of each hospital was diminished by two shillings. To how many hospitals did he give ?

### Answers.

No. 19. DOUBLE ACROSTIC.

| | | |
|---|---|---|
| H | ospital | S |
| O | lympi | A |
| S | al | T |
| P | er | U |
| I | lcheste | R |
| T | allar | D |
| A | stræ | A |
| L | yl | Y |

Correct answers have been received from Gip, Gretta, Ostrich, Peeler, Ellice.

No. 20. MATHEMATICAL QUESTION.

The only number which satisfies the condition is 7, thus :—

| | |
|---|---|
| First person had | $3\frac{1}{2}+\frac{1}{2}=4$ |
| Second „ | $1\frac{1}{2}+\frac{1}{2}=2$ |
| Third „ | $\frac{1}{2}+\frac{1}{2}=1$ |
| | Total 7. |

Correct answers have been received from Peeler, Ostrich, A. M. E., May, H. J. C.. Condorcet, Gretta, Mater, Gip, Mr. J. High, K. M., Ellice, Mrs. Bedingfeld.

Answers must be addressed to THE PRIZE EDITOR, AMUSEMENTS WITH PROFIT, THE HOSPITAL, Norfolk House, Norfolk Street, Strand, W.C., not later than the *first* post on Saturday next. The full name and address must in all cases be given, but a *nom de plume* may be added for publication. Only one side of the paper should be written on. The "Amusements with Profit" Coupon (see advertisement pages) must be enclosed with replies.

